# Hypocalcemia and Vitamin D Deficiency in Children with Inflammatory Bowel Diseases and Lactose Intolerance

**DOI:** 10.3390/nu13082583

**Published:** 2021-07-28

**Authors:** Martyna Jasielska, Urszula Grzybowska-Chlebowczyk

**Affiliations:** Department of Pediatrics, Faculty of Medical Sciences, Medical University of Silesia in Katowice, 40752 Katowice, Poland; urszulachlebowczyk@wp.pl

**Keywords:** lactose intolerance, inflammatory bowel disease, hypocalcemia, vitamin D

## Abstract

Background: A diet restricted in dairy products can cause calcium and vitamin D deficiency and, secondarily, lead to malnutrition and low bone mass. The aim of the study was to determine the incidence hypocalcemia and vitamin D deficiency in children with inflammatory bowel diseases and lactose intolerance (LI). Material and Methods: A total of 107 patients were enrolled to the study (mean age 14.07 ± 3.58 years; 46.7% boys): 43 with Crohn’s disease (CD), 31 with ulcerative colitis (UC), and 33 with functional abdominal pain (AP-FGID). Hydrogen breath test with lactose and laboratory tests to assess the calcium-phosphate metabolism were performed in all patients. The results of densitometry were interpreted in 37 IBD patients. Results: LI was diagnosed in 23.2% patients with CD, 22.6% with UC, and 21.2% children with AP-FGID, (*p* = 0.9). Moreover, 9.5% patients with CD, in 21.4% with UC, and in 51.5% with AP-FGID had optimal concentration of 25(OH)D (*p* = 0.0002). Hypocalcemia was diagnosed in 21% of patients with CD, 16.1% with UC patients, AP-FGID patients had normal calcium levels (*p* = 0.02). There was no difference in concentrations of total calcium, phosphorus, and 25(OH)D between patients on low-lactose diet and normal diet (*p* > 0.05). BMD Z-score ≤ −1 SD was obtained by 12 CD patients (48%), and 6 with UC (50%). Conclusion: The use of a low-lactose diet in the course of lactose intolerance in children with inflammatory bowel diseases has no effect on the incidence of calcium-phosphate disorders and reduced bone mineral density.

## 1. Introduction

The mean age of diagnosis of inflammatory bowel diseases (IBD) in children (around 12 years of age) is the time of the greatest increase in bone mass, therefore, these patients are particularly at risk of lowering the peak bone mass in relation to their healthy peers [1]. The main factors that can affect growth and bone metabolism are malnutrition, delayed puberty, decreased physical activity, and chronic inflammatory process (e.g., participation of pro-inflammatory cytokines IL-6, TNFα, IL-1ß) [2]. Bone mass is also decreased by the use of glucocorticosteroids, which directly cause osteoblast dysfunction and accelerate osteoblast apoptosis and reducing calcium absorption from the gastrointestinal tract and increasing renal calcium excretion (stimulating parathyroid hormone release in the parathyroid glands) [2]. Puberty (Tanner stages 2, 3, 4) has been shown to be the most sensitive period in which glucocorticoid use may lead to irreversible bone loss [2,3].

On the other hand, IBD patients, including pediatric patients, are at risk of vitamin D deficiency. Serum vitamin D levels above 15 ng/mL are found only in 15–34% of children with IBD [2]. Maintaining the optimal vitamin D level in patients with inflammatory bowel diseases is of great importance not only for proper bone mineralization, but also due to its immunomodulatory effect. Since the vitamin D receptor is also found in immune system cells, the influence of vitamin D on IBD has been the subject of many studies. The immunomodulatory effect of vitamin D reduces the proliferation of T helper lymphocytes and the production of pro-inflammatory cytokines (mainly IL-6, Il-12, IL-23), and increases the number of regulatory lymphocytes T [4]. All of this indicates that vitamin D deficiency and hypocalcemia have many clinical implications in IBD patients. In Poland, as in most Western countries, milk and its products are the main source of calcium in the diet—as much as 30% of calcium from milk and dairy products is absorbed. In addition, dairy products are also a source of protein, phosphorus, potassium, and vitamins A, D, B2 (riboflavin), and B12 (cobalamin) [5]. Multiple observational studies show that consumers who avoid milk have lower bone mineral density (BMD) and a 2.7-fold higher risk of bone fractures in pre-pubertal children compared to individuals with higher milk consumption [3,6,7,8].

In our previous study, we showed that the incidence of SNP of a promoter region of LCT gene, lactose malabsorption, and intolerance does not differ among children with inflammatory bowel diseases from the population [9]. Only 1/3 of patients with lactose intolerance developed adaptation and returned to a normal diet in the 3 years of observation [10]. Therefore, it is extremely important to know the prevalence of vitamin D deficiency and hypocalcemia, as well as the factors influencing these deficiencies in IBD patients on a low-lactose diet.

### Aim

The aim of the study was to determine the prevalence of hypocalcemia and vitamin D deficiency in children with inflammatory bowel diseases and lactose intolerance.

## 2. Methods

A total of 107 patients were enrolled in the study at the Department of Gastrology, Upper Silesian Children’s Health Centre, Katowice in 2016–2017 (mean age 14.07 ± 3.58, median 15.6 years; 46.7% were boys). The study population was formed by three groups: Crohn’s disease (CD, *n* = 43), ulcerative colitis (UC, *n* = 31), functional abdominal pain (AP-FGID, *n* = 33). The diagnosis of IBD was based on the modified Porto criteria [11]. 15 patients (20.2%) were newly diagnosed with IBD. The AP-FGID was diagnosed on the basis of IVth Rome criteria [12].

IBD patients were evaluated for the clinical expression of the disease, the location of the disease (the Paris classification), and the disease activity (PCDAI/PUCAI) [13,14,15]. Disease duration was calculated from the date of the first diagnostic endoscopy.

A hydrogen breath test (HBT) was performed in all patients. A Gastrolyzer Gastro+ (SynecPOL, Poland) was used to measure hydrogen concentration in exhaled air. Measurements were carried out every 30 min for 3 h after oral ingestion of lactose (1 g lactose/kg body weight, maximum 25 g). Lactose intolerance (LI) was diagnosed, if in any of the measurements the hydrogen concentration in the exhaled air increased ≥20 ppm over the baseline value and clinical symptoms occurred (ex. abdominal pain, diarrhea). A fasting blood sample and daily second urine sample were obtained from all subjects. Plasma parathormon (PTH), vitamin D (25[OH]D), calcium (Ca), phosphate (P), alkaline phosphatase (AP), magnesium (Mg), and albumin were measured from both patients and controls by standard methods. Native parathyroid hormone was measured by an electrochemiluminescent immunometric assay (Cobas), the reference range was 15–65 pg/mL. Serum 25-hydroxyvitamin D (25[OH]D) was assessed by electrochemilumi- nescent method (Elecsys Vitamin D total, Cobas). Serum 25(OH)D ranges of 30–50 ng/mL were considered optimal, 20–30 ng/mL were suboptimal. A concentration below 20 ng/mL was considered a vitamin D deficiency.

Urine calcium, phosphate, and creatinine were tested on the same day on the second fasting sample of urine. Calcium-creatinine (Ca/Cr) and phosphate-creatinine (P/Cr) index were calculated from the obtained results.

Areal bone mineral density (BMD, g/cm^2^) of the whole body was measured with DXA Lunar Prodigy Primo (GE Healthcare, Chicago, IL, USA). The reference values for children were pediatric program standards adjusted to age and ethnicity (NHANES/Lunar).

Statistical analysis was conducted in licensed MedCalc Statistical Software version 17.7 (MedCalc Software bvba, Ostend, Belgium; 2017). The character of the distribution of quantitative variables was verified by the Shapiro–Wilk test. The evaluation of intergroup differences for quantitative variables was based on Student’s *t* test (or ANOVA) or Mann–Whitney U test (or Kruskall–Walis). Intergroup differences for categorical variables were evaluated by chi-square test or Fisher’s exact test. *p* < 0.05 was considered statistically significant.

The consent of the Bioethics Committee of Silesian Medical University for the medical experiment was obtained, No. KNW/00/KB1/49/I/15 dated 16 June 2015. Patients and their guardians provided informed consent. In this group of patients, another study was performed in which the incidence of lactose intolerance, lactose malabsorption and SNP polymorphisms related to hypolactasia were estimated [9].

## 3. Results

### 3.1. Location and Activity of IBD

The mean duration of CD and UC was similar and was respectively: 21.27 months (median 13 months, range 1–108 months) and 21.72 months (median 12 months, range 0.5–96 months). All patients were classified based on Paris classification [13]; see Table 1.

The distribution of disease activity assessed by PCDAI or PUCAI did not differ in the IBD groups (*p* > 0.05). In patients with Crohn’s disease, mild disease was diagnosed in 18 patients (41.9%), moderate in 20 (46.5%), and severe in 5 (11.6%). Among patients with UC, mild disease was diagnosed in 14 children (45.2%), similarly to the moderate disease—14 children (45.2%), a severe one was diagnosed only in 2 children (6.4%). In one child with UC the disease was inactive (3.2%).

### 3.2. Therapy

In the six months prior to study enrolment, 17 patients (39.5%) with CD were treated with total enteral nutritional treatment (EEN). Oral glucocorticoids (GCS) in the last 3 months were received by 35.5% of patients with UC and 9.3% of patients with CD. Azathioprine was taken by a similar percentage of children with CD and UC (39.5% and 38.7%, respectively). In patients with CD infliximab was used more often than with UC (21% vs. 6.4%).

### 3.3. Clinical Characteristics

As many as 93.6% patients with UC and almost 70% with CD had normal body weight, while 16.3% of children with CD were diagnosed with body weight deficiency. Body weight above 1 SD for age and sex was diagnosed in 14% of children with CD, 6.4% of UC and 27.3% of AP-FGID patients. The differences in the incidence of weight disorders were not statistically significant (*p* = 0.2).

### 3.4. Laboratory Tests

Detailed results of the laboratory tests are presented in Table 2

### 3.5. Vitamin D

The optimal concentration of the 25(OH)D in serum (>30 ng/mL) was found only in 9.5% of CD patients and 21.4% of UC patients. However, in the AP-FGID group, the concentration of 25(OH)D > 30 ng/mL was achieved by more than half of the patients; the difference was statistically significant (*p* = 0.0002). At the same time, vitamin D deficiency (concentration < 20 ng/mL) was most often diagnosed in patients with CD (29 children, 69.1%), less often in patients with UC (13 children, 46.4%), and in the AP-FGID group only in 18, 2% of children (6 children); this difference was also statistically significant (*p* = 0.0001).

Patients with CD and UC supplemented vitamin D with the same frequency as patients with AP-FGID (69.7%, 61.3% and 77.4% respectively, *p* = 0.36). Patients with AP-FGID used lower doses of vitamin D (median 600 IU, IQR 500–800 IU) than patients with CD (median 800 IU, IQR 700–1000 IU) and UC (median 1000 IU, IQR 400–1000 IU), but the difference was not statistically significant (*p* = 0.6). Additionally, patients who were tested in the spring and summer months (May—September) had significantly higher serum levels of vitamin D than the patients tested in the autumn or winter (October–April, *p* = 0.02).

In the group of patients with CD, vitamin D deficiency was significantly more frequent in patients with isolated involvement of the upper gastrointestinal tract or small intestine, proximal to 1/3 of the distal ileum (L4a, L4b in the Paris classification), in relation to the involvement of the terminal ileum and large intestine (L1–L3), as well as lesions involving different sections of the gastrointestinal tract (L4a/L4b and L1–L3, *p* = 0.002).

Among patients from all groups, no correlation was found between the serum concentration of 25(OH)D and age, sex, BMI Z-score, disease duration, other parameters of calcium-phosphate metabolism, and occurrence of lactose intolerance and low-lactose diet (*p* > 0.05). In addition, there was no correlation between the concentration of vitamin D and the duration of the disease, the number of exacerbations and disease activity (measured on the PCDAI/PUCAI scale), as well as the treatment used (*p* > 0.05) among patients with IBD.

### 3.6. Calcium

Hypocalcemia, i.e., serum total calcium concentration lower than the reference value for age, was diagnosed in 21% of patients with Crohn’s disease (9 children) and 16.1% of patients with ulcerative colitis (5 children). All patients with AP-FGID had total calcium levels within the normal range. The difference in the incidence of calcium deficiency between the groups was statistically significant (*p* = 0.02). Table 2. Total calcium level was significantly lower in the study group, both in total (*p* = 0.0007) and separately—in UC and CD, than with AP-FGID (*p* = 0.003). Patients with newly diagnosed CD had a lower total calcium serum concentration than patients with longest disease duration (median 2.33 mmol/l vs. 2.38 mmol/L), and this difference was statistically significant (*p* = 0.04).

It is worth noticing that the concentration of ionized calcium did not differ between the studied groups (*p* = 0.8). In a multiple regression analysis in the studied group of children with UC, the concentration of total calcium in the serum was influenced by the use of glucocorticosteroids in the last 3 months (*p* = 0.009, R^2^ = 0.5), while among the studied children with CD it was serum total protein concentration (*p* = 0.0009, R^2^ = 0.24). The concentration of total calcium in the serum of patients with IBD did not depend on age, sex, localization and behavior of the disease, treatment (other than glucocorticosteroids) and serum vitamin D concentration. (*p* > 0.05).

### 3.7. Others

The median Ca/Cr ratio was similar in each group; it was 0.06 and 0.08 mg/mg in CD and UC, respectively, and 0.05 mg/mg in the AP-FGID group (*p* = 0.9), refer to Table 2. Hypercalciuria (Ca/Cr > 0.21 mg/mg) was found in 4 children with inflammatory bowel diseases (5.4%) and 1 with AP-FGID (3%). Patients with CD treated with EEN in the last six months prior to enrolment in the study had significantly more often higher calcium-creatinine indexes (hypercalciuria was found in 3 patients) than patients without this treatment (*p* = 0.01). In the multiple regression analysis, the following factors influenced the Ca/Cr ratio in patients with CD: BMI Z-score (*p* = 0.01) and nutritional treatment in the last six months (*p* < 0.0001, R^2^ = 0.36).

### 3.8. Lactose Intolerance

LI was diagnosed in 10 patients with CD (23.2%), 7 with UC (22.6%) and 7 patients with AP-FGID (21.2%, *p* = 0.9). All children diagnosed with LI were initially on a lactose-free diet, and then, after the symptoms of intolerance relieved, a low-lactose diet (depending on symptoms). Low lactose diet was considered as consumption 12 g of lactose (or less) a day. The median duration of the diet in patients with Crohn’s disease was 10 months, and 24 months with ulcerative colitis. 4 patients with lactose intolerance followed a low-lactose diet for a long time (more than three years).

LI had no effect on total calcium levels in any of the groups (*p* > 0.05). There was also no statistically significant difference in the concentrations of the other studied parameters of calcium-phosphate metabolism between patients with lactose intolerance—following a lactose-free/low diet, and without intolerance—not using an elimination diet (*p* > 0.05). Table 3.

### 3.9. DXA

Thirty-seven patients with IBD (25 with CD and 12 with UC) had total body without the head densitometry (TBLH DXA). The mean BMD Z-score in children with IBD was −0.67 ± 1.02 SD (median −0.8 SD). BMD Z-scores were similar among children with CD and UC. The suboptimal bone mass (BMD Z-score ≤ −1 SD) was obtained by 12 patients with CD (48%), and 6 patients with UC (50%). In contrast, low bone mass (BMD Z-score ≤ −2 SD) was found in 8% children with CD and 8% with UC (respectively 2 and 1 patient, *p* = 0.9).

Growth retardation (G1, Paris Classification) was found in 12 patients, and in 9 of them the bone mineral density was <−1 SD. Median BMD Z-score in patients with growth retardation was −1.3 SD and was lower in relation to children with normal growth (median −0.65 SD, *p* = 0.06). Analyzing the studied group, it was found that there was no difference in BMD Z-score among patients with lactose intolerance and lactose tolerant patients (*p* = 0.7). There was no important history of bone fracture in patients with IBD and no patient met the criteria for the diagnosis of osteoporosis.

## 4. Discussion

Patients with inflammatory bowel diseases are at high risk of vitamin and micronutrient deficiency and bone mineralization disorders. A diet restricted in dairy products carries a risk of insufficient calcium intake in the diet. The consumption of milk and milk products is important not only because of the high calcium content, but also the presence of lactose, which facilitates the absorption of calcium in gut [5,6,16]. It is an unresolved issue whether the supply of calcium in people with digestive disorders/lactose intolerance is sufficient. DiStefano et al., when examining patients with lactose malabsorption and intolerance, found that patients with LI consume statistically significantly less calcium in the diet than lactose tolerant patients [17]. On the other hand, Medeiros et al. comparing the daily intake of calcium in people with lactose digestive disorders, lactose intolerance, and healthy controls found no difference [18]. The majority of calcium absorption studies show that neither dietary lactose nor lactase deficiency in healthy adults has a significant impact on calcium absorption [19]. In our study, lactose intolerance had no effect on the results of laboratory tests assessing calcium and phosphate metabolism both with IBD and with AP-FGID. There was no difference in the concentration of total calcium in the serum between patients with lactose intolerance (using low-lactose diet) and children with normal diet. Also, there was no difference in serum concentrations of the vitamin D between these groups. These results are consistent with those available in the world literature that shows that vitamin D deficiency is not a feature of LI in prepubertal children [19].

Vitamin D deficiency (concentration < 20 ng/mL) was found in 70% of patients with CD, almost 50% of patients with UC, and nearly 20% of children with AP-FGID, the difference was statistically significant. A greater prevalence of vitamin D deficiency in children with IBD than with AP-FGID has been confirmed by numerous studies so far [1,2]. In contrast, in study conducted in Seoul, the incidence of vitamin D deficiency in IBD patients was not significantly different from that in patients with functional abdominal pain [20]. Adolescent IBD patients are predisposed to serum vitamin D deficiency due to limited sun exposure, decreased absorption of vitamin D in the intestine due to inflammatory lesions or bowel resection, and incorrect or too low dose of supplemented vitamin D [1,20]. The concentration of vitamin D in patients with CD was influenced by the month in which patient were enrolled to the study (higher concentrations were recorded in the spring and summer), isolated location of lesions in the upper gastrointestinal tract and small intestine (lower concentration), as well as vitamin D supplementation—patients supplementing vitamin D had a higher concentration of 25(OH)D in the serum than patients without supplementation. In the study, vitamin D supplementation was performed by 69% of patients with CD and 61% of patients with UC. This is a high result compared to the study by Hartman et al., where it was estimated that only 40% of IBD patients supplemented vitamin D [21]. A systematic review of over two-hundred trials featured diverse treatment regimens that were predominantly insufficient in correcting vitamin D deficiency or maintaining adequate levels in children with IBD [22].

In the study group, hypocalcemia was found in 21% of patients with CD and 16.1% of patients with UC, which was significantly more frequent than in the AP-FGID group, where all patients had normal serum total calcium levels. The mean concentration of total calcium in the serum of patients with IBD was also lower than in the AP-FGID group. Similar results were presented by other authors, who proved that the concentration of total calcium in the serum of IBD patients is lower than in patients with FIGD [18,20]. In the study group, lower levels of total calcium in the serum of UC patients were reported in patients using glucocorticoids in the last 3 months. In contrast, in patients with CD, serum total calcium was positively correlated with serum total protein. On the other hand, it has been proven that patients with CD are exposed to inadequate dietary calcium intake—as many as 79% take less than 80% of the daily calcium requirement, and only 19% supplement with calcium [21]. In a study by Lopes et al., only 16% of patients with IBD satisfied the daily calcium requirement [23].

One of the interesting conclusions from the conducted study is the presence of lower concentrations of total calcium in patients with newly diagnosed CD. The literature data previously reported this fact, e.g., in the Cho study, serum calcium levels in newly diagnosed CD patients were lower than in UC patients or controls. According to the authors, low serum micronutrient concentrations are associated with a generalized inflammatory reaction in the body of patients, which we cannot confirm, as our study did not assess inflammatory markers [21].

The Ca/Cr ratio were similar in children from the study and AP-FGID groups. In the group of patients with CD treated with EEN, in the last six months the Ca/Cr ratio achieved was significantly higher. It should be noted that the concentration of total calcium in the serum of patients treated with EEN was also higher (however, the difference was not statistically significant). In the study by Pappa et al., hypercalciuria was found in approximately 8% of patients with IBD, regardless of the dose of vitamin D administered, which is more often than in the general population [24]. In contrast, Laakso et al. found a higher Ca/Cr ratio in patients with IBD than in healthy controls, but the authors explained this difference with the cumulative dose of glucocorticosteroids received by patients that increase urinary calcium excretion [25]. In the study group, patient therapy had no significant effect on the Ca/Cr ratio in urine, however, in this study the cumulative dose of glucocorticoids was not assessed.

The concentrations of other parameters—alkaline phosphatase activity, serum inorganic phosphorus, and parathyroid hormone concentrations did not differ significantly between IBD patients and the AP-FGID group, which is consistent with the current knowledge [20,21].

Despite the similar concentration of inorganic phosphorus in the serum in all groups of patients, the P/Cr ratio was significantly lower in the group of children with IBD than in the AP-FGID, which is different from the previous reports in the literature. Due to the increased risk of urolithiasis, especially stones composed of calcium phosphates and calcium oxalates, the excretion of electrolytes in the urine has been the subject of many studies so far. In a study by Buno et al., it was proven that the urinary excretion of phosphorates in adult patients with CD was increased in relation to the control [26].

Disturbances in calcium and phosphate homeostasis, especially calcium deficiency, have many clinical implications, including disturbances in bone mineralization. In the study group, half of the IBD patients who underwent bone densitometry obtained suboptimal and low bone mineral density (TBLH BMD Z-score ≤ −1 SD). The recent study showed that the prevalence of suboptimal BMD among pediatric patients with IBD was 33.3% (spine BMD) and 31% (total-body BMD) and the prevalence of low BMD was 12.5% (spine BMD) and 27% (total-body BMD) [27]. However, bone mineralization disorders in the population of children with inflammatory bowel diseases occur with different frequency, e.g., Sigurdsson et al. estimated the incidence of reduced bone mineral density (BMD ≤ −1 SD) at 16%, and Laakso at 23% [25,28].

In the study group, 8% of children with CD and 8% with UC had low bone mineral density (BMD Z-score ≤ −2 SD), a lower result compared to the literature data [27]. Among children in the study group, low bone mineral density was similarly common among children with CD and UC. Sylvester et al. showed that 43% of patients with CD and 39% of UC patients had BMD < −1 SD at the time of diagnosis [1].

Patients with IBD are at increased risk of decreased bone mass. Poor nutritional status, delayed maturation, decreased physical activity, as well as chronic inflammation and malabsorption disorders are recognized as factors affecting bone growth and metabolism [2,27,29]. In the studied group of patients with CD, lower values of BMD Z-score were noted among patients with delayed growth (patients in the G1 group of the Paris classification). A positive correlation was also found between age and height and BMD Z-score, which is related to the fact that bone mineral density increases with age until it reaches its peak bone mass in the third decade of life (however, it is at the limit of statistical significance). There was no correlation between bone mineral density and the activity, location, or duration of the disease (both in CD and UC).

The negative effect of glucocorticosteroids on bone mineral density is well documented [2,23,29]. In the studied group, no dependence on the use of glucocorticosteroids on the BMD Z-score was found, this may be due to the small study group (only 8 patients with densitometry were taking glucocorticoids before entering the study), and the cumulative dose of glucocorticosteroids was not calculated.

Both in the discussed study and in the available literature on the pediatric IBD population, no correlation was found between the vitamin D and the TBLH BMD Z-score [20]. On the other hand, in adults with IBD, there are reports linking these two parameters—patients with short-term disease and low serum 25(OH)D concentrations had significantly lower bone mineral density, and in control studies, an increase in vitamin D metabolite concentration was associated with an increase in BMD [30].

Disturbances in the parameters of calcium and phosphate metabolism can be reflected in disturbances in bone mineralization [7]. Both IBD alone and the dairy-restricted diet are considered risk factors for bone mineralization disorder [2,3,7]. In the analyzed group of patients, the results of bone mineral density measurements in lactose-intolerant patients on a low-lactose/lactose-free diet were similar to those obtained in lactose-tolerant patients and not on an elimination diet. These studies are also consistent with other publications, e.g., Medeiros et al., where results of the bone mineral density examination of the lumbar spine were similar in patients with lactose intolerance/lactose malabsorption and without these disorders [18].

To our knowledge, this is the first study that simultaneously assesses the incidence of lactose intolerance in pediatric patients with inflammatory bowel diseases and the prevalence of calcium-phosphate disorders as well as bone mineral density. A significant limitation of the study is the small size of the study group, especially patients diagnosed with lactose intolerance, which limits the possibility of inferring calcium and phosphate metabolism disorders in this subgroup. Among patients with inflammatory bowel diseases, not all patients have currently performed densitometry. Although this was due to the organizational reasons for the study, it made it difficult to assess the frequency of low BMD in the group of patients with IBD. An equally important limitation of the study was the lack of assessment of the cumulative dose of glucocorticoids among patients with inflammatory bowel diseases, it was only taken into account whether the patients had received these drugs in the last 3 months prior to enrolment in the study. The development of knowledge about the frequency of calcium and phosphate disorders, their determinants, and risk factors in patients with inflammatory bowel diseases may bring significant implications in the diagnostic and therapeutic process of patients, contributing to the prevention of future complications of the disease and the improvement of the patients’ quality of life.

## 5. Conclusions

Patients with CD and UC, regardless of the presence of lactose intolerance, significantly more often have vitamin D deficiency and hypocalcemia. The use of a low-lactose diet in the course of lactose intolerance in children with inflammatory bowel diseases has no effect on the incidence of calcium-phosphate disorders and reduced bone mineral density.

## Figures and Tables

**Table 1:** Paris classification.

	Symbol	Description	N (%)
Crohn’s disease			
Age of diagnosis	A1a	<10 years	6 (14%)
	A1b	10–17 years	35 (81.4%)
	A2	17–40 years	2 (4.6%)
	A3	>40 years	
Location	L1	Distal 1/3 ileum (±limited cecal disease)	20 (46.5%)
	L2	Colonic	6 (13.9%)
	L3	Ileocolonic	13 (30.3%)
	L4a	Upper disease proximal to ligament of Treitz	12 (27.9%)
	L4b	Upper disease distal to ligament of Treitz and proximal to distal 1/3 ileum	6 (13.9%)
Behavior	B1	Nonstricturing nonpenetrating	31 (72.1%)
	B2	Stricturing	9 (20.9%)
	B3	Penetrating	3 (7%).
	*p*	Perianal disease modifier	7 (16.3%)
Growth	G0	No evidence of growth delay	31 (72.1%)
	G1	Growth delay	12 (27.9%)
Ulcerative colitis			
Extent	E1	Ulcerative proctitis	6 (19.4%)
	E2	Left-sided UC (distal to splenic flexure)	10 (32.2%)
	E3	Extensive (hepatic flexure distally)	6 (19.4%),
	E4	Pancolitis (proximal to hepatic flexure)	9 (29%)
Severity	S0	Never severe	24 (77.4%)
	S1	Ever severe	7 (22.5%)

**Table 2:** The results of calcium and phosphate metabolism parameters determined from the serum and urine in the studied group.

Parameter		CD	UC	AP-FGID	*p*
Ca (mmol/L)	Medium ± SD	2.35 ± 0.12	2.35 ± 0.1	2.42 ± 0.08	0.003
	Median	2.38	2.35	2.43	
	IQR	2.29–2.43	2.31–2.41	2.39–2.46	
Ca^++^ (mmol/L)	Medium ± SD	0.99 ± 0.09	0.98 ± 0.1	0.99 ± 0.07	0.8
	Median	1.01	1.03	1.01	
	IQR	0.94–1.97	0.91–1.05	0.93–1.06	
P (mmol/L)	Medium ± SD	1.4 ± 0.23	1.4 ± 0.2	1.44 ± 0.24	0.7
	Median	1.39	1.36	1.37	
	IQR	1.23–1.50	1.21–1.58	1.25–1.66	
PTH (pg/mL)	Medium ± SD	30.7 ± 9.3	29.4 ± 11.2	29.3 ± 9.0	0.5
	Median	30.8	28.4	27.1	
	IQR	24.2–34.5	21.9–33.6	21.6–35.5	
25(OH)D (ng/mL)	Medium ± SD	17.3 ± 10.4	23.3 ± 10.2	29.3 ± 8.7	0.00003
	Median	15.1	21.9	28.7	
	IQR	10–24.3	4.5–29.4	22.4–36.4	
AP (U/L)	Medium ± SD	134.4 ± 55.7	160 ± 83.8	183.1 ± 109.5	0.276
	Median	122	151.5	206	
	IQR	98–156	104–203	71.75–262.7	
Total protein (g/L)	Medium ± SD	71.2 ± 6.8	68.8 ± 18.9	72.8 ± 3.5	0.2
	Median	70.6	73.1	73.3	
	IQR	67.6–75.1	69.9–75.8	70.3–75.5	
Mg (mmol/L)	Medium ± SD	0.86 ± 0.05	0.84 ± 0.06	0.86 ± 0.06	0.2
	Median	0.84	0.85	0.86	
	IQR	0.81–0.88	0.8–0.87	0.83–0.9	
Ca/Cr (mg/mg)	Medium ± SD	0.1 ± 0.09	0.1 ± 0.1	0.08 ± 0.07	0.9
	Median	0.06	0.08	0.05	
	IQR	0.03–0.14	0.03–0.12	0.03–0.12	
P/Cr (mg/mg)	Medium ± SD	0.4 ± 0.28	0.48 ± 0.28	0.6 ± 0.28	0.03
	Median	0.34	0.43	0.66	
	IQR	0.22–0.51	0.31–0.66	0.36–0.8	

IQR—Inter quartile ratio.

**Table 3:** Test results of calcium and phosphate metabolism parameters depending on lactose tolerance in the studied groups.

		CD		UC		AP-FGID	
		LI (+)	LI (−)	LI (+)	LI (−)	LI (+)	LI (−)
Ca (mmol/L)	Medium ± SD	2.37 ± 0.09	2.34 ± 0.13	2.35 ± 0.08	2.35 ± 0.1	2.42 ± 0.08	2.42 ± 0.08
	Median	2.39	2.38	2.34	2.37	2.4	2.43
	IQR	2.3–2.43	2.26–2.44	2.3–2.39	2.32–2.42	2.33–2.52	2.4–2.45
P (mmol/L)	Medium ± SD	1.30 ± 0.39	1.38 ± 0.33	1.43 ± 0.19	1.4 ± 0.2	1.44 ± 0.24	1.46 ± 0.25
	Median	1.32	1.4	1.33	1.38	1.28	1.4
	IQR	1.24–1.44	1.23–1.55	1.21–1.59	1.21–1.58	1.15–1.58	1.28–1.68
25(OH)D (ng/mL)	Medium ± SD	18.1 ± 12.1	16 ± 10.4	23.4 ± 9.1	23.3 ± 10.2	29.3 ± 8.7	29.4 ± 8.5
	Median	17.8	14.6	27.6	21.4	26	33.2
	IQR	9.6–27.9	10–21.3	13.4–30.1	16–29.46	19.6–28.6	22.4–37.4
PTH (pg/mL)	Medium ± SD	26.8 ± 13.3	30 ± 10.7	30.5 ± 11.7	29.4 ± 11.2	29.3 ± 9	30.7 ± 9
	Median	32.1	30.6	30.2	26.4	29.1	23.6
	IQR	22.6–36.7	24.8–34.3	28.2–36.2	21–31	27.2–39.8	21.5–35.4
Ca/Cr (mg/mg)	Medium ± SD	0.08 ± 0.08	0.09 ± 0.1	0.09 ± 0.1	0.1 ± 0.1	0.08 ± 0.07	0.08 ± 0.07
	Median	0.06	0.06	0.04	0098	0.047	0.055
	IQR	0.03–0.2	0.04–0.13	0.027–0.07	0.039–0.12	0.01–0.154	0.04–0.102
P/Cr (mg/mg)	Medium ± SD	0.23 ± 0.12	0.41 ± 0.31	0.47 ± 0.24	0.43 ± 0.3	0.6 ± 0.28	0.64 ± 0.28
	Median	0.28	0.36	0.43	0.43	0.58	0.62
	IQR	0.21–0.34	0.24–0.56	0.28–0.86	0.32–0.66	0.5–0.714	0.35–0.88

## Data Availability

The statistical analysis and database used to support the findings of this study may be released upon application to the Medical University of Silesia, Department of Pediatrics, who can be contacted by the corresponding author.
